# A bibliometric and visual analysis of research on echinocandin treatment for urinary tract infections based on VOSviewer and CiteSpace

**DOI:** 10.3389/fcimb.2026.1891369

**Published:** 2026-09-22

**Authors:** Fangqing Xie, Yan Zhou, Chun Liu, Qinchuan Li, Yun Lu, Hua Wei, Lu Zhou, Xirui Guo, Shihao Yan, Shibo Lin, Ya Zou

**Affiliations:** Department of Clinical Pharmacology, West China School of Medicine, Sichuan University, Sichuan University Affiliated Chengdu Second People’s Hospital, Chengdu Second People’s Hospital, Chengdu, China

**Keywords:** antifungal resistance, bibliometric analysis, candiduria, Citespace, echinocandins, urinary tract infection, VOSviewer

## Abstract

**Background:**

Echinocandins have emerged as potential therapeutic options for urinary tract infections (UTIs), particularly involving multidrug-resistant fungi. Given the fragmented nature of the evidence and the lack of comprehensive synthesis, this study aimed to systematically evaluate the global research landscape, collaboration networks, and emerging hotspots in this field through a bibliometric analysis.

**Methods:**

Publications related to echinocandin therapy for UTIs from database inception to March 20, 2026 were retrieved from PubMed and Web of Science. This bibliometric review was conducted and reported in accordance with the BIBLIO (Preliminary Guideline for Reporting Bibliometric Reviews of the Biomedical Literature) minimum requirements. VOSviewer (1.6.20) was used for collaboration network and keyword co-occurrence analysis. CiteSpace (6.4.R1) was used for burst detection, timeline visualization, and cluster analysis. Additional bibliometric indicators including total citations, average citations per publication, H-index, citation density, and identification of influential journals were calculated.

**Results:**

A total of 47 publications were included, with peaks in 2016 and 2025 (six each). The limited number of publications reflects the emerging nature of this research field and the ongoing debate regarding the role of echinocandins in UTI management. The United States and Spain were leading contributors. Keyword analysis revealed a shift from early themes toward multidrug-resistant pathogens, particularly Candida auris, Candida glabrata, and multidrug resistance. Cluster analysis identified eight significant clusters Q = 0.755, S = 0.9196, focusing on candidate drugs, pathogen identification, and antifungal resistance mechanisms.

**Conclusion:**

Research on echinocandins for UTIs remains limited but shows sustained interest. The field has evolved toward multidrug-resistant fungal pathogens. Lack of robust clinical evidence remains a major barrier. Future studies should prioritize prospective clinical investigations and pharmacokinetic/pharmacodynamic evaluations.Robust prospective clinical studies and PK/PD evaluations remain essential to establish definitive therapeutic role. We advocate for updated, evidence-based stewardship protocols that clearly define indications for echinocandin use in fluconazole-refractory UTIs.

## Introduction

Urinary tract infection (UTI) is among the most common infectious diseases encountered in clinical practice. Fungal UTIs, predominantly caused by Candida species, have increased substantially in recent years, with candiduria affecting up to 40% of hospitalized patients (Geerlings, 2016; Maleb et al., 2020; Castellano-Sánchez et al., 2025). Notably, the rising prevalence of non-albicans Candida species—particularly the multidrug-resistant pathogens Candida auris and Candida glabrata—has posed substantial challenges to current antifungal management strategies (Sulaiman et al., 2014; Wolfgruber et al., 2024).

Echinocandins, including caspofungin, micafungin, anidulafungin, and the recently developed long-acting agent rezafungin, constitute an important class of antifungal agents that exert fungicidal activity through noncompetitive inhibition of β-(1,3)-D-glucan synthase, thereby disrupting fungal cell wall synthesis (Thompson et al., 1999; Gajdács et al., 2019; Garcia-Effron, 2020). Owing to their favorable safety profile, potent antifungal activity, and limited drug interactions, echinocandins are currently recommended as first-line therapy for invasive candidiasis, including candidemia (Wagner et al., 2006; Huang et al., 2025). However, their role in UTIs remains debated due to conventional pharmacokinetic concerns: echinocandins exhibit high molecular weights, extensive protein binding, and minimal renal excretion (e.g., <0.1% for anidulafungin), leading current Infectious Diseases Society of America (IDSA) guidelines to discourage routine use for candiduria (Sobel et al., 2007; Azanza Perea, 2016; Epelbaum et al., 2025).

Nevertheless, accumulating clinical evidence—including case reports, case series, and prospective studies—has challenged this traditional view, demonstrating favorable outcomes with echinocandin therapy in pyelonephritis, complicated UTIs, and infections caused by resistant Candida species (Pappas et al., 2016; Rezai et al., 2017; Rkieh et al., 2022). Pharmacodynamic parameters, such as a urinary Cmax/MIC ratio >4 for micafungin, have been proposed as predictors of therapeutic success, suggesting that tissue penetration rather than urinary excretion may drive efficacy in upper UTIs (Biagi et al., 2019). This discrepancy between guideline endorsement and emerging evidence, together with the development of next-generation echinocandins like rezafungin and the continuous evolution of antifungal resistance (Grau et al., 2016; Bassetti et al., 2025; Forrister et al., 2025), underscores the urgent need for a systematic assessment of the research landscape.

Despite its growing clinical relevance, the literature on echinocandin therapy for UTIs remains fragmented, consisting primarily of heterogeneous case reports, small case series, and isolated pharmacokinetic studies. No comprehensive synthesis has systematically characterized the knowledge structure, developmental trajectory, collaboration networks, or evolving research priorities of this field (Cornely et al., 2025). Traditional narrative reviews, while valuable, are inherently subjective and cannot quantitatively map scientific evolution or detect emerging trends. Bibliometric analysis offers a robust, objective, and reproducible quantitative framework to address these gaps—enabling the visualization of scientific collaboration networks, identification of knowledge foundations, detection of research hotspots, and prediction of future directions (Abdelwahab et al., 2025). Among available tools, VOSviewer excels at visualizing co-authorship and keyword co-occurrence networks, while CiteSpace offers distinct advantages in burst detection and temporal trend analysis (Lian et al., 2025; Cristea et al., 2026). To our knowledge, this study represents the first bibliometric visualization analysis focusing specifically on echinocandin therapy for UTIs. By mapping the global research landscape and identifying evolving hotspots, we aim to clarify developmental trajectories, reveal knowledge gaps, and provide evidence-based insights to guide future clinical investigations, stewardship protocols, and therapeutic decision-making.

## Materials and methods

### Literature search strategy

A comprehensive literature search was conducted in the PubMed and Web of Science databases to identify publications related to echinocandin therapy for urinary tract infections (UTIs), from database inception to March 20, 2026. PubMed and Web of Science were selected for their comprehensive coverage of high-impact biomedical journals and their established roles as primary data sources for bibliometric analyses using VOSviewer and CiteSpace. For Web of Science, we employed Topic search (covering Title, Abstract, and Keywords) to maximize the capture of relevant studies where UTI-related terms appeared in keywords even if not in the title or abstract.

The search strategy combined controlled vocabulary terms and free-text keywords related to echinocandins (including caspofungin, micafungin, anidulafungin, rezafungin, and ibrexafungerp) with terms associated with UTIs, including urinary tract infection, candiduria, pyelonephritis, cystitis, bacteriuria, and catheter-associated urinary tract infection. Detailed search strategies for both databases are provided in the **Supplementary Materials** (**Supplementary Table 1**).

### Eligibility criteria

Publications were considered eligible if they met the following criteria:a)Published in peer-reviewed English-language medical journals; b)Focused on the therapeutic application of echinocandins for UTIs; c)Included original research articles, case reports, case series, or review articles relevant to the study topic.

The exclusion criteria were as follows:a)Conference abstracts, editorials, news reports, books, patents, and duplicate publications; b)Articles clearly unrelated to echinocandin therapy for UTIs; c)Publications lacking essential bibliographic information, including author names, publication year, or institutional affiliations.

### Data collection and analysis

Two researchers performed literature deduplication and screening. If there was disagreement, a third researcher made the judgment to ensure that the included literature conformed to the research topic. Finally, 47 articles were included. This bibliometric review was conducted and reported in accordance with the BIBLIO (Preliminary Guideline for Reporting Bibliometric Reviews of the Biomedical Literature) minimum requirements (Montazeri et al., 2023); the completed checklist is provided in **Supplementary Table 2**. The selected literature was exported in plain text format with full records and cited references, and analyzed using Excel 2021, CiteSpace (version 6.4.R1) and VOSviewer (version 1.6.20). VOSviewer (version 1.6.20) was used for co-authorship network analysis of countries, institutions, authors, and keyword co-occurrence analysis (**Supplementary Table 2**). CiteSpace (version 6.4.R1) was used for journal, author, reference co-citation analysis and timeline visualization. Additional bibliometric indicators, including total citations, average citations per publication, H-index, citation density (average citations per year), and identification of influential journals and highly cited references, were calculated using Excel and CiteSpace. Microsoft Excel 2021 was used for basic statistics and trend fitting (**Figure 1**).

**Figure 1:**
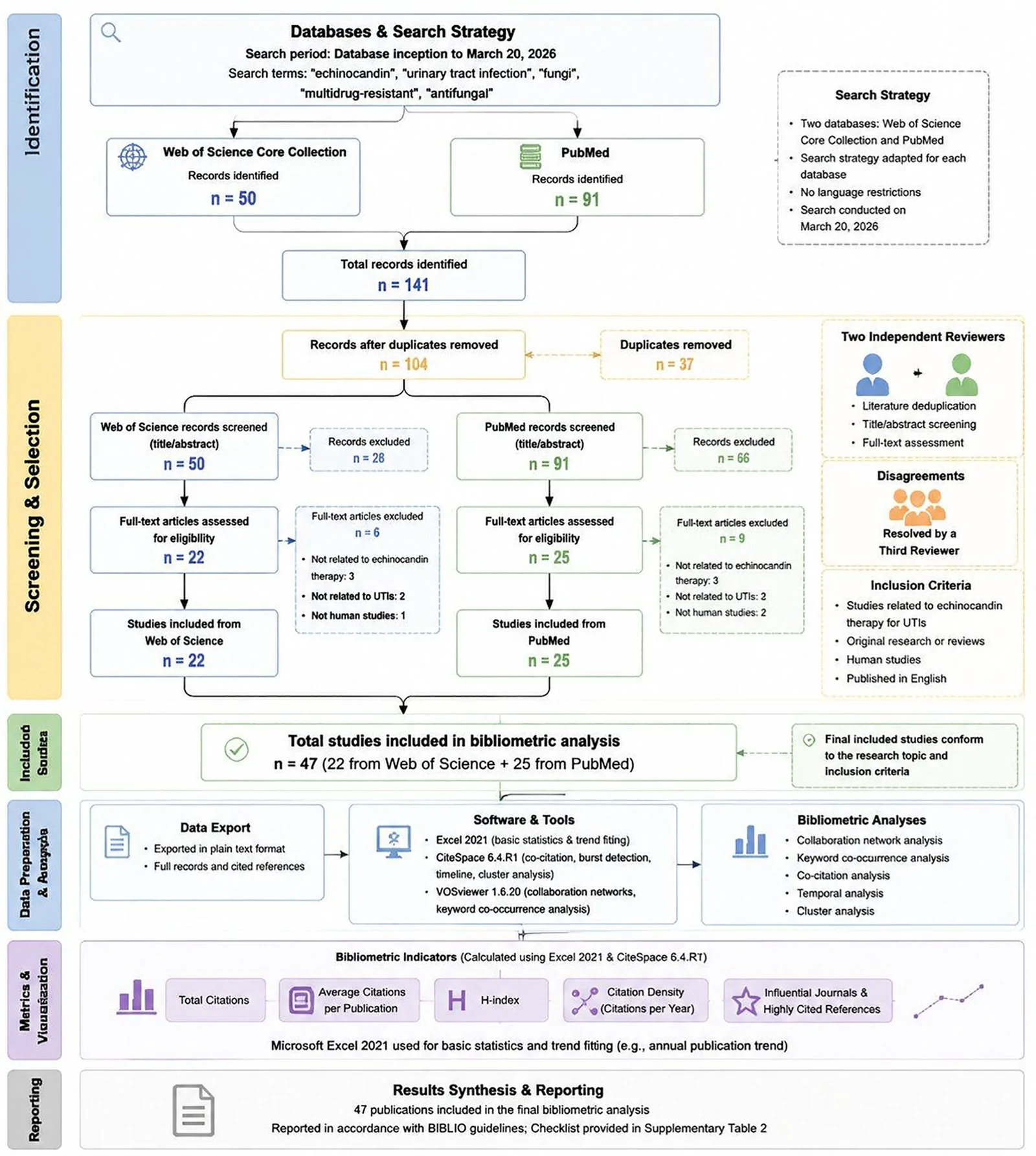
Flowchart of literature screening for studies on echinocandins for UTIs.

## Results

### Annual publication trends

Annual publication output serves as a key indicator for evaluating developmental trends within a research field, providing insight into research activity, thematic evolution, and disciplinary maturity (Hassan and Duarte, 2024). After screening and deduplication, 47 publications on echinocandin therapy for UTIs were identified from database inception through March 20, 2026. As shown in **Figure 2**, annual publication output remained modest overall, with notable peaks observed in 2016 and 2025, each yielding six publications. In the remaining years, annual output fluctuated between zero and five publications. This relatively stable yet limited publication pattern suggests that echinocandin therapy for UTIs remains a niche research area, while the persistence of scholarly output indicates ongoing clinical and academic interest, particularly in response to emerging challenges in antifungal resistance.

**Figure 2:**
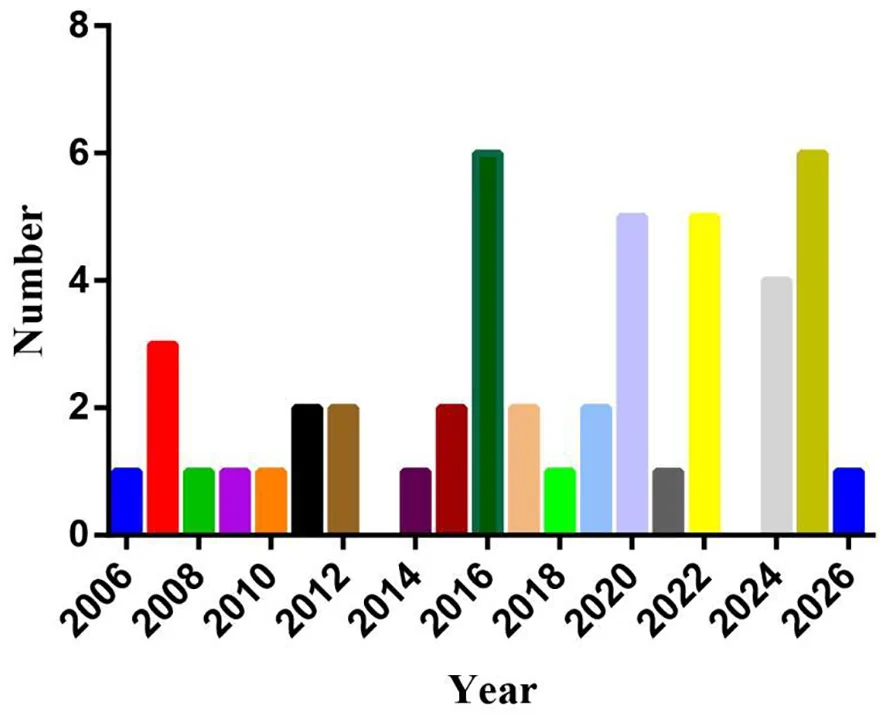
Annual publication trend of echinocandins for UTIs.

### Publication type distribution

Among the 47 included publications, the distribution of document types was as follows: original research articles n=15 (31.9%), case reports n=14 (29.8%), case series n=10 (21.3%), and review articles n=8 (17.0%). This heterogeneity indicates that the evidence base is primarily composed of clinical observations and summaries, with a smaller proportion of controlled studies. It is also important to note that the heterogeneity of publication types may have influenced citation-based indicators. Although review articles constituted only 17.0% (8/47) of the included publications, they tend to accumulate citations more rapidly and in greater numbers than original research articles or case reports. Consequently, the high citation counts observed for certain authors and institutions (as presented in **Tables 1**, **2**) may partly reflect their involvement in highly cited review articles rather than a broader research output in this field. This potential bias should be considered when interpreting citation-based rankings.

**Table 1:** Top 10 institutions ranked by citation impact in echinocandin-UTI research.

Rank	Institution	Country	Publications	Total citations	Average citations/publication	Year
1	Medical College of Georgia	USA	1	152	152	2011
2	University of Michigan	USA	1	152	152	2011
3	Wayne State University	USA	1	152	152	2011
4	University of Texas at San Antonio	USA	1	52	52	2020
5	University of Illinois System	USA	1	26	26	2020
6	Hospital del Mar	Spain	1	24	24	2016
7	Institut Hospital del Mar d’Investigacions Mèdiques	Spain	1	24	24	2016
8	Instituto de Salud Carlos III	Spain	1	24	24	2016
9	Laboratori de Referència de Catalunya	Spain	1	24	24	2016
10	Universitat Autònoma de Barcelona	Spain	1	24	24	2016

Ranking is based on total citation counts as a measure of academic impact, not publication productivity. The “Average Citations per Publication” column provides a normalized indicator of citation intensity per article. Institutions with identical citation counts are ranked alphabetically.

**Table 2:** Top 10 authors ranked by citation impact in echinocandin-UTI research.

Rank	Author	Publications	Citations	Average citations/publication
1	Fisher, John F	1	152	152
2	Kauffman, Carol A	1	152	152
3	Newman, Cheryl A	1	152	152
4	Sobel, Jack D	1	152	152
5	Chaturvedi, Ashok K	1	52	52
6	Dinakaran, Hemamalini	1	52	52
7	Lopez-Ribot, Jose L	1	52	52
8	Thomas, Derek P	1	52	52
9	Uppuluri, Priya	1	52	52
10	Griffith, Nicole	1	26	26

Ranking is based on total citation counts as a measure of scholarly impact, not publication count. *Grau, S. is included as a collaborative hub with multiple publications; authors with identical citation counts are ranked alphabetically. Due to the limited dataset (n=47), the majority of top-cited authors contributed single publications.

### Country and institutional contributions

The 47 included publications involved 36 institutions worldwide.Institutions with the highest citation frequencies included Medical College of Georgia, University of Michigan, and Wayne State University (**Table 1**), indicating their substantial academic influence in this field.

Institutional collaboration network analysis generated by VOSviewer identified six major clusters. Notably, strong collaborative relationships were observed among Hospital del Mar, Institut Hospital del Mar d’Investigacions Mèdiques, and Instituto de Salud Carlos III, reflecting active regional research cooperation, particularly within Spain (**Figure 3**).

**Figure 3:**
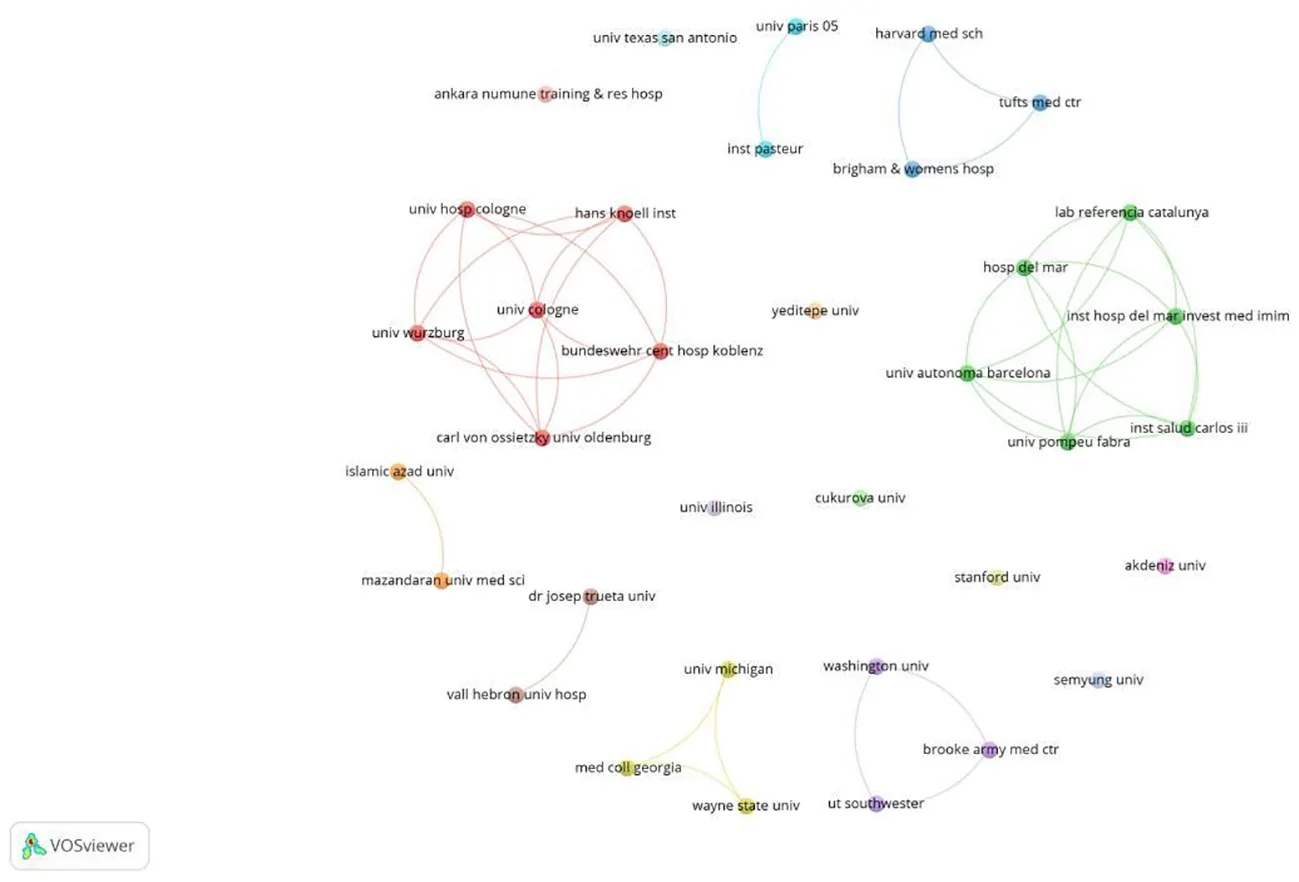
Institutional collaboration network for echinocandins for UTI research. Nodes represent individual institutions; node size is proportional to the number of publications from each institution. Edges represent collaborative relationships between institutions; edge thickness indicates the strength of collaboration (i.e., number of co-authored publications). Institutions are grouped into six distinct clusters, each denoted by a different color, representing regional or thematic research groups. The network was generated using VOSviewer (version 1.6.20) with a minimum publication threshold of one document per institution. Notably, Spanish institutions (Hospital del Mar, Institut Hospital del Mar d’Investigacions Mèdiques, and Instituto de Salud Carlos III) formed a tightly connected cluster, reflecting active regional research cooperation.

### Author publications and collaboration

A total of 47 articles involved 60 authors. Among the top 10 most cited authors, Fisher, John F. and three other authors shared the highest citation counts, followed by Chaturvedi, Ashok K. and three additional authors (**Table 2**). As illustrated in **Figure 4**, the author collaboration network comprised multiple researchers from diverse institutions, reflecting well-established collaborative relationships within this field. The node-link structure revealed a clear core–periphery pattern, with authors such as Grau, S. and Charlier, Caroline emerging as key collaborative hubs through their close connections with other researchers.

**Figure 4:**
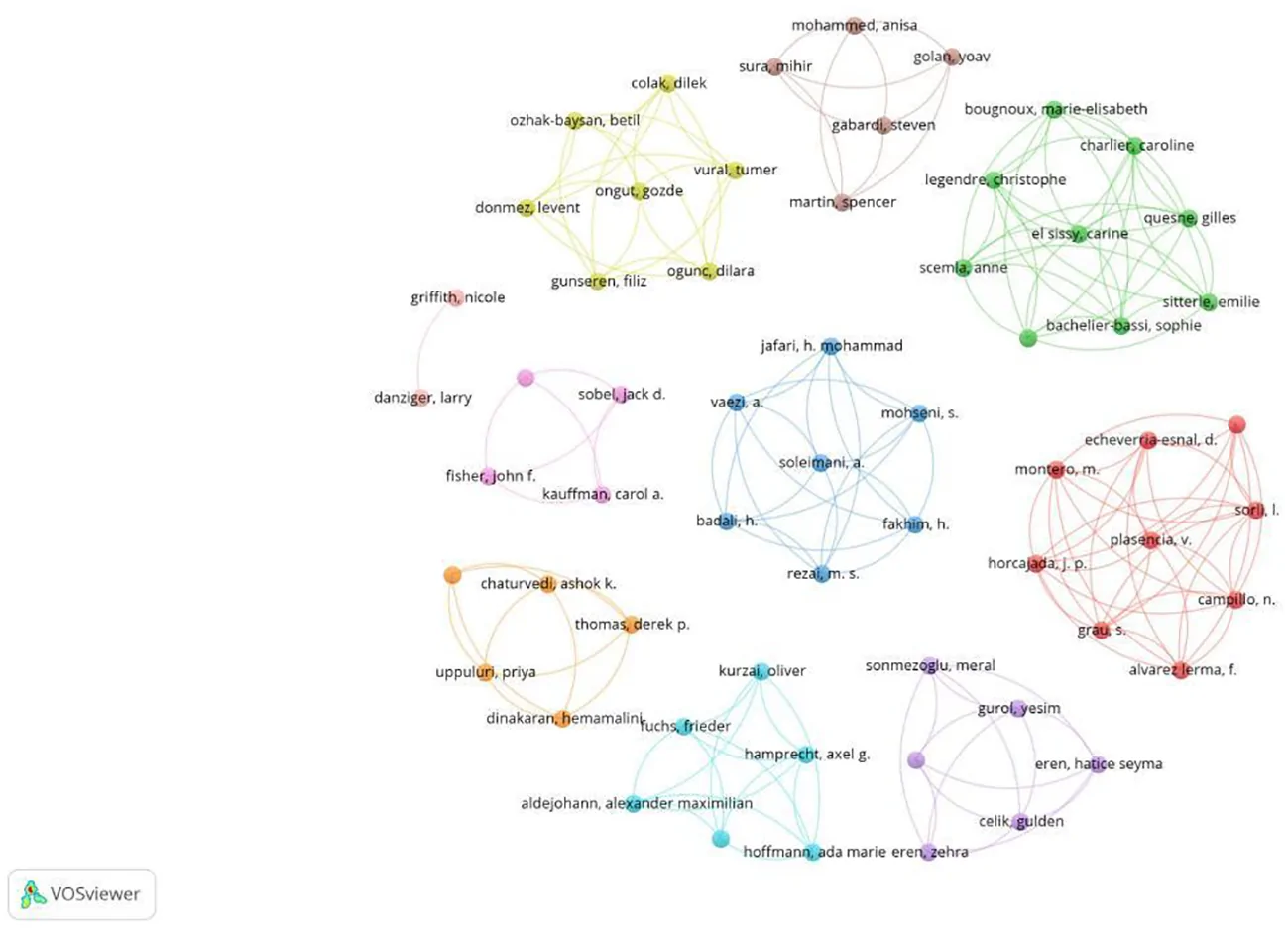
Author collaboration network for echinocandins for UTI research. Nodes represent individual authors; node size is proportional to the number of publications per author. Edges represent co-authorship relationships; edge thickness reflects collaboration frequency. Authors are grouped into multiple clusters by color, with a clear core–periphery structure. Key collaborative hubs, such as Grau, S. and Charlier, Caroline, are visible as central nodes with dense connections to other researchers. The network was generated using VOSviewer with a minimum publication threshold of one document per author.

### Research hotspots identified by keyword co-occurrence analysis

Using VOSviewer with a minimum keyword co-occurrence threshold of two, a total of 36 keywords were identified and incorporated into the co-occurrence network analysis (**Figure 5**). In this network, larger node sizes represent higher keyword frequency, reflecting greater research attention within the field.

**Figure 5:**
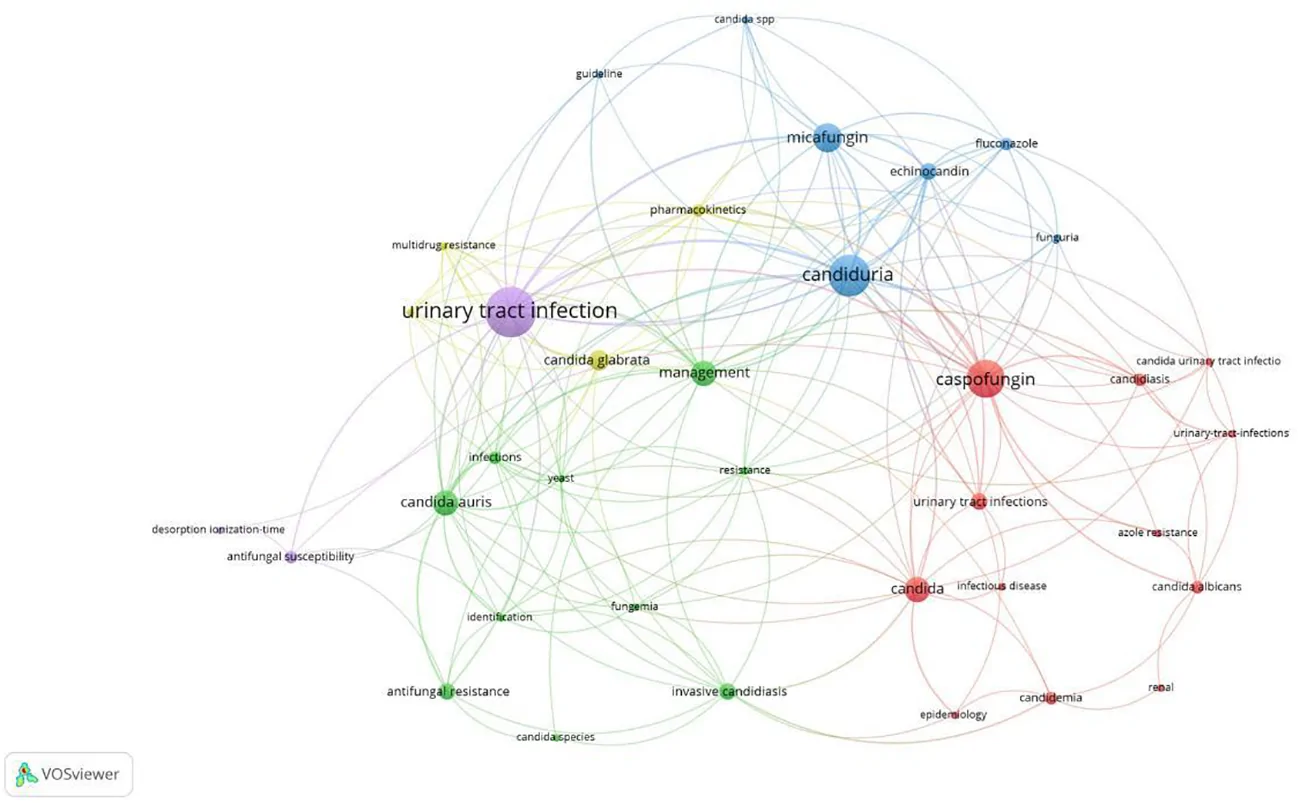
Keyword co-occurrence network for echinocandins for UTI research. Nodes represent keywords that appeared at least twice in the included publications; node size is proportional to the frequency of keyword occurrence. Edges represent co-occurrence relationships between keywords; edge thickness indicates the strength of association. Keywords are grouped into thematic clusters denoted by different colors. The network was generated using VOSviewer with a minimum co-occurrence threshold of two. The most frequent keywords—”urinary tract infection,” “candiduria,” and “caspofungin”—appear as the largest nodes, reflecting their central role in the research field.

As shown in **Table 3**, urinary tract infection, candiduria, and caspofungin emerged as the three most frequently occurring keywords, indicating that these topics have constituted the primary research focus in studies investigating echinocandin therapy for UTIs.

**Table 3:** Top 20 keywords in echinocandins for UTI research.

Rank	Keyword	Frequency	Rank	Keyword	Frequency
1	urinary tract infection	12	11	invasive candidiasis	4
2	candiduria	10	12	urinary tract infections	4
3	caspofungin	9	13	antifungal susceptibility	3
4	micafungin	7	14	candida albicans	3
5	candida	6	15	candidemia	3
6	candida auris	6	16	candidiasis	3
7	management	6	17	fluconazole	3
8	candida glabrata	5	18	infections	3
9	antifungal resistance	4	19	pharmacokinetics	3
10	echinocandin	4	20	amphotericin-b	2

Temporal overlay visualization further demonstrated a notable evolution in research emphasis. Prior to 2018, studies were predominantly centered on caspofungin and candiduria, reflecting early interest in therapeutic feasibility and clinical application. Since 2018, the focus has progressively shifted toward broader UTI-related themes, suggesting an expansion of research interest from individual antifungal agents toward more comprehensive investigations of disease management and therapeutic strategies (**Figure 6**).

**Figure 6:**
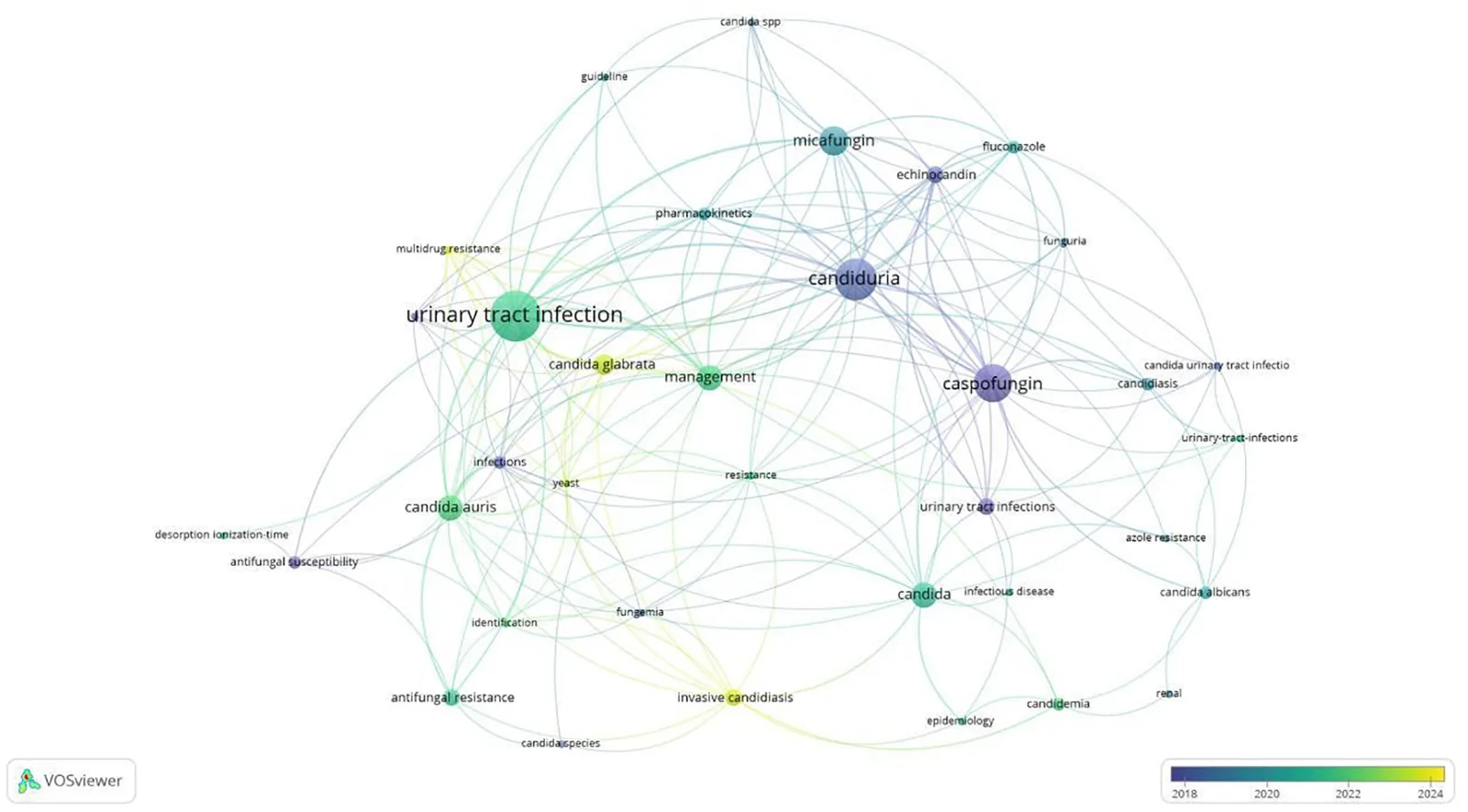
Keyword timeline for echinocandins for UTI research. This image presents the same keyword co-occurrence network as **Figure 5**, but with nodes colored according to the average year of publication of the documents in which each keyword appears. The color gradient is interpreted as follows:Dark blue nodes: Keywords with an average occurrence year ≤ 2016, representing early research foci (e.g., “caspofungin,” “candiduria”).Green nodes: Keywords with an average occurrence year between 2017 and 2021, representing intermediate-term research themes (e.g., “antifungal resistance,” “management”).Yellow/bright nodes: Keywords with an average occurrence year ≥ 2022, representing recently emerging research hotspots (e.g., “Candida auris,” “Candida glabrata,” “multidrug resistance”).This temporal overlay allows readers to intuitively track the evolution of research priorities over time, from early feasibility assessments of echinocandins in candiduria toward contemporary concerns about multidrug-resistant fungal pathogens. The visualization was generated using VOSviewer’s overlay visualization module.

### Temporal evolution of research themes: burst detection and timeline visualization

To comprehensively characterize the temporal dynamics of research foci, we employed two complementary analytical approaches: keyword burst detection (which identifies sudden surges in keyword frequency) and timeline visualization (which maps the persistence and cluster affiliation of research themes over time). These two methods address distinct but related questions: burst analysis reveals frontier shifts (i.e., which topics gained rapid attention during specific periods), while timeline visualization illuminates the long-term structural evolution of the knowledge base (i.e., which foundational themes persisted and how new branches emerged).

Keyword burst analysis (**Figure 7**) identified 14 keywords with statistically significant frequency surges over the study period. During the early stage (approximately 2016–2019), bursts were concentrated on foundational epidemiological and diagnostic themes, including “urinary tract infections,” “candidiasis,” and “fungal infections.” In the intermediate stage (approximately 2017–2022), bursts shifted toward more refined clinical investigations, emphasizing specific pathogens such as “Candida albicans,” therapeutic management strategies, and conventional antifungal agents like “fluconazole.” In the current stage (approximately 2020 to present), the strongest bursts have centered on multidrug-resistant fungal pathogens—notably “Candida auris” and “Candida glabrata”—alongside rapid identification technologies and mechanisms underlying multidrug resistance. This temporal sequence demonstrates a clear progression from disease recognition and therapeutic feasibility toward complex resistance challenges.

**Figure 7:**
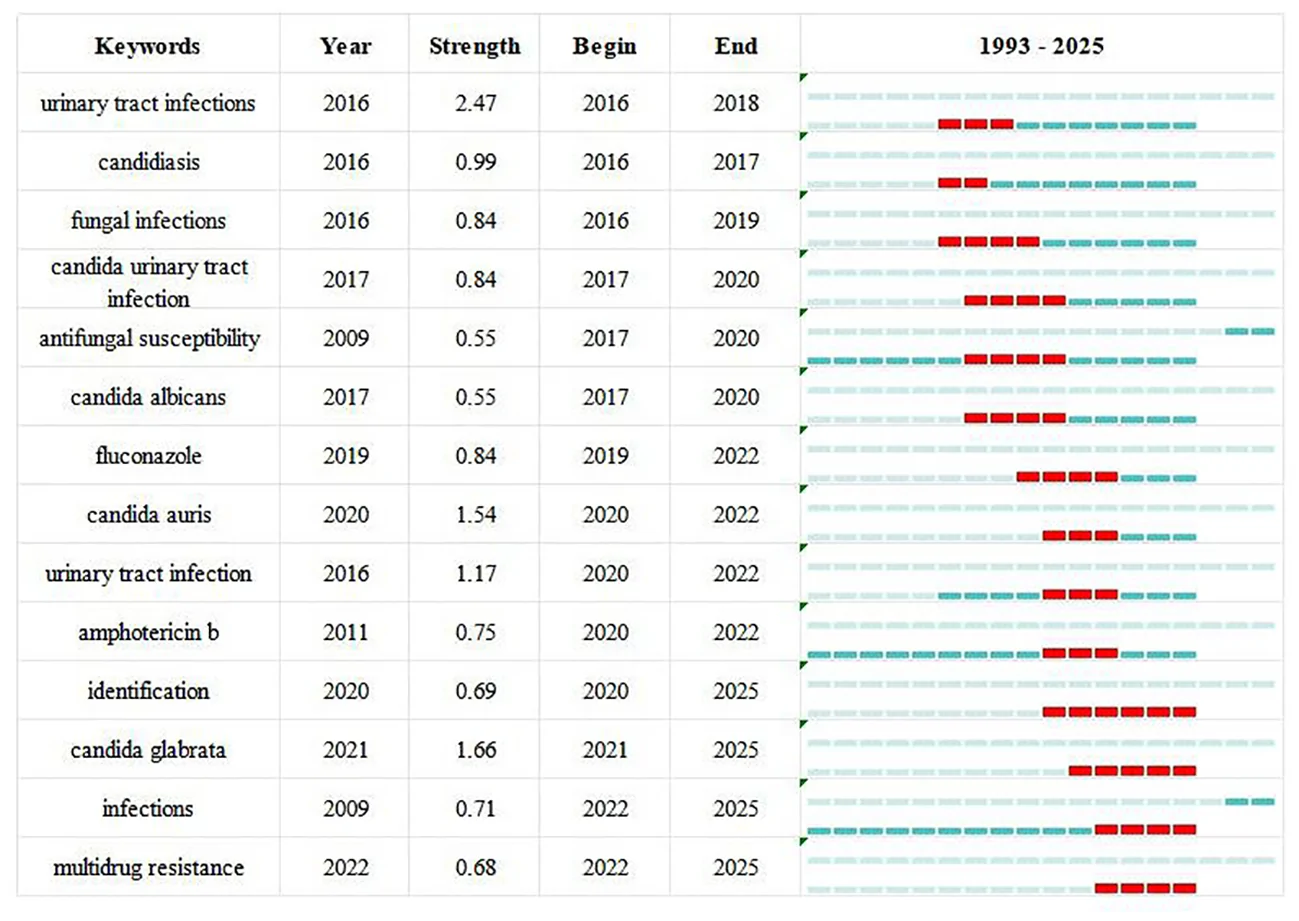
Top 14 keywords with the strongest citation bursts in echinocandins for UTI research. The image was generated using CiteSpace (version 6.4.R1) burst detection algorithm. Each horizontal bar represents a keyword, with the red segment indicating the time interval during which the keyword experienced a statistically significant surge in frequency. The “Year” column denotes the first year the keyword appeared in the dataset; “Strength” indicates the burst intensity (higher values represent more abrupt increases in frequency); and “Begin/End” mark the start and end years of the burst period. This analysis identifies rapidly emerging research interests, such as “Candida auris” (burst from 2020 to 2026), reflecting shifting research frontiers toward multidrug-resistant pathogens.

Timeline visualization (**Figure 8**) complements the burst analysis by illustrating how these keywords are distributed across thematic clusters and how long they have persisted within the research landscape. Core keywords such as “urinary tract infection” and “candiduria” spanned the entire study period, reflecting sustained clinical interest irrespective of temporal bursts. Among pathogenic species, “Candida albicans,” “Candida auris,” and “Candida glabrata” appeared as the primary organisms of concern, with the latter two exhibiting both strong burst intensity (**Figure 7**) and sustained presence across recent years (**Figure 8**). Among echinocandins, “caspofungin” remained the most extensively investigated agent, maintaining a stable presence throughout the timeline, while newer agents such as “rezafungin” appeared only in the most recent period, suggesting an emerging but not yet mature research trajectory. Taken together, the burst analysis captures the dynamic frontier shifts driven by clinical urgency (e.g., emerging outbreaks of C. auris), whereas the timeline visualization reveals the stable intellectual backbone of the field, highlighting foundational topics that continue to underpin clinical practice.

**Figure 8:**
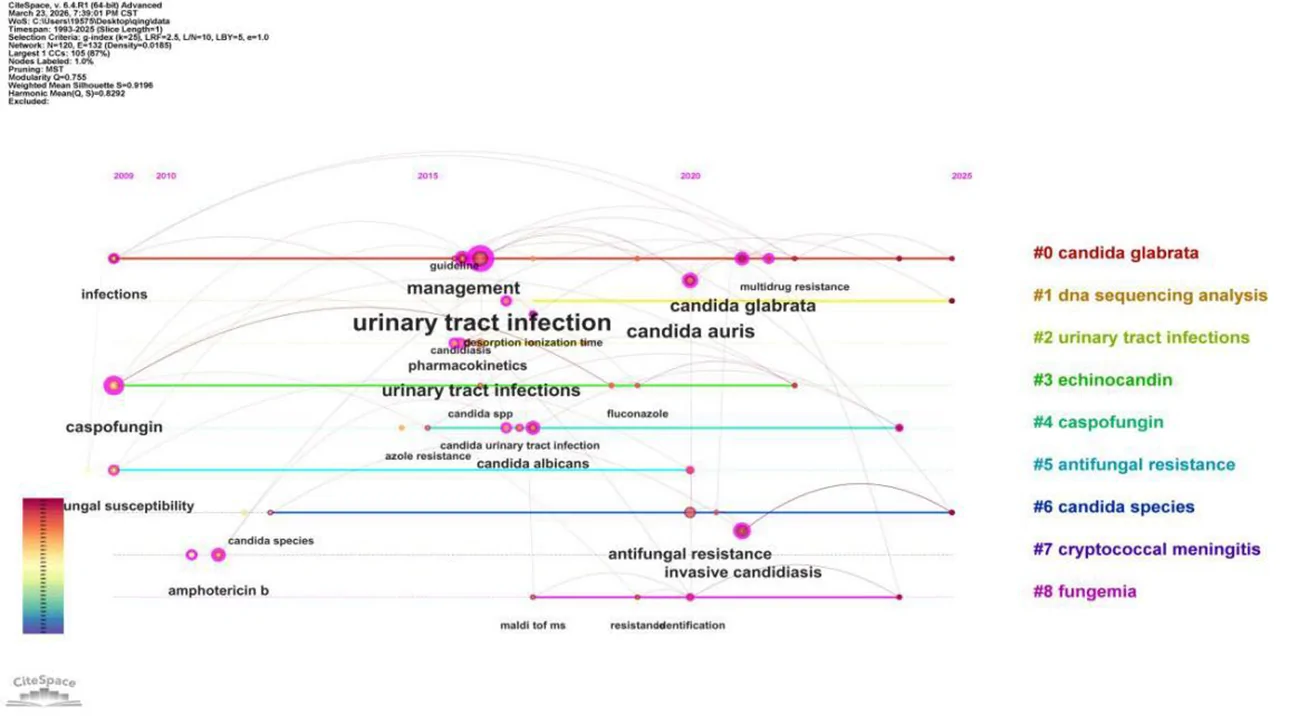
Keyword timeline view for echinocandins for UTI research. This image was generated using CiteSpace’s timeline view, which clusters keywords by thematic categories (vertical axis) and displays their temporal distribution across the study period (horizontal axis, from 2006 to 2026). Each node represents a keyword; node size is proportional to frequency. Keywords within the same cluster are positioned on the same horizontal line, with their positions along the x-axis indicating the year of first appearance. Core keywords that persist across the entire study period (e.g., “urinary tract infection,” “candiduria”) appear as large nodes spanning multiple years, reflecting sustained clinical interest, while newer topics (e.g., “Candida auris”) appear in more recent years, indicating emerging research directions.

### Keyword cluster analysis

Keyword cluster analysis groups semantically related terms to reveal the knowledge structure and thematic distribution within a research field. In this study, keyword clustering analysis was conducted using the log-likelihood ratio (LLR) algorithm in CiteSpace to identify the major research themes in echinocandin therapy for urinary tract infections (UTIs) (**Figure 9**).

**Figure 9:**
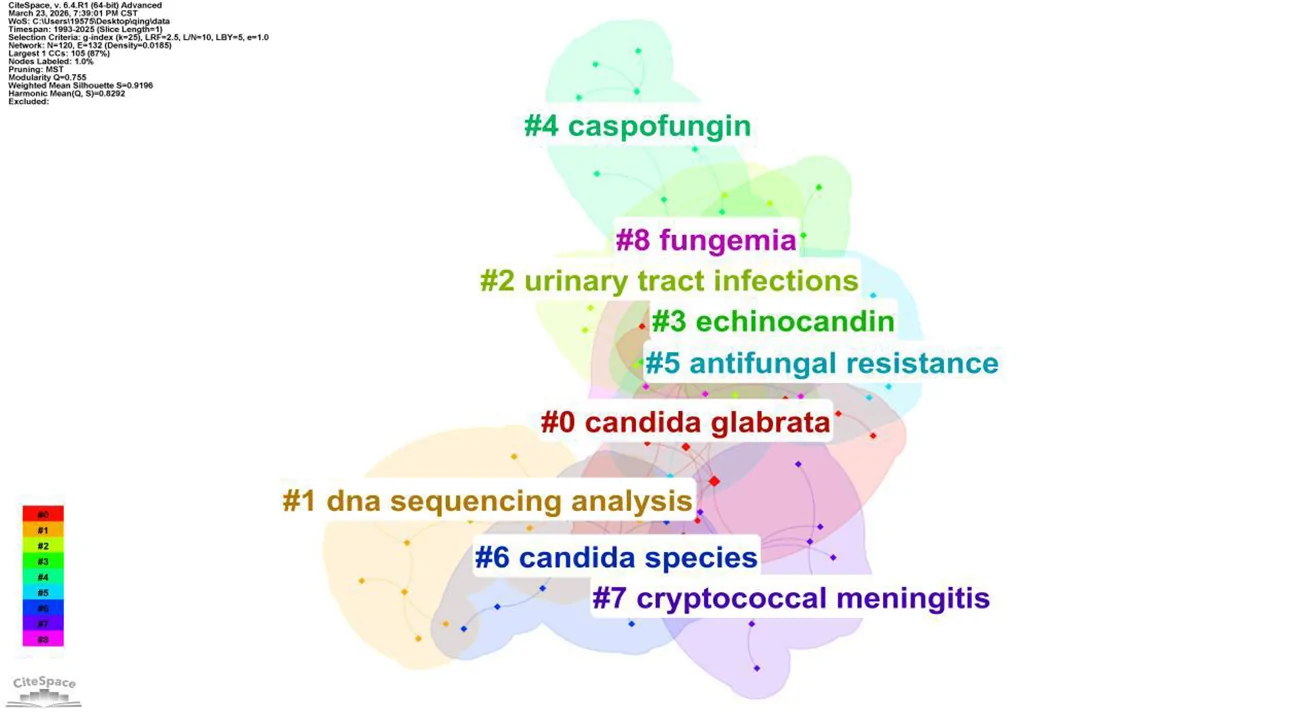
Keyword cluster network for echinocandins for UTI research. This image presents the same keyword co-occurrence data as **Figure 5**, but with keywords grouped into statistically significant clusters using the log-likelihood ratio (LLR) algorithm in CiteSpace. Each cluster is labeled with its most representative term and denoted by a distinct color. The clustering quality is supported by modularity Q = 0.755 and mean silhouette S = 0.9196, indicating a well-structured and highly consistent network. A total of eight meaningful clusters were identified, including “Candida glabrata,” “DNA sequencing analysis,” “urinary tract infections,” “echinocandin,” “caspofungin,” “antifungal resistance,” “Candida species,” and “cryptococcal meningitis.” These clusters reflect two major thematic domains: pathogen-oriented investigations (left side) and antifungal therapeutic research (right side).

The clustering results demonstrated strong statistical reliability, with a modularity value of Q = 0.755 and a mean silhouette score of S = 0.9196, indicating a well-structured and highly consistent clustering network. A total of eight meaningful keyword clusters were identified:Candida glabrata, DNA sequencing analysis, urinary tract infections, echinocandin, caspofungin, antifungal resistance, Candida species, and cryptococcal meningitis.

These clusters reflected two major thematic domains: pathogen-oriented investigations and antifungal therapeutic research. Specifically, pathogen-focused studies emphasized fungal identification, molecular diagnostic approaches, and species-specific epidemiology, whereas therapeutic research concentrated on echinocandin efficacy, antifungal resistance mechanisms, and clinical treatment optimization. Among these, caspofungin emerged as the most prominent drug-centered research focus, highlighting its central role in this field.

### Co-citation analysis and landmark publications

We performed a co-citation analysis using CiteSpace to identify the intellectual foundation of the field. The most frequently co-cited references included the IDSA Clinical Practice Guideline for the Management of Candidiasis (Pappas et al., 2016), the pivotal pharmacokinetic study by Bellmann et al (Bellmann and Smuszkiewicz, 2017), and the MSG-01 trial (Ostrosky-Zeichner et al., 2014). These landmark publications established the clinical guideline framework, the pharmacokinetic rationale for echinocandin use, and the safety profile in high-risk patients, respectively. The co-citation network revealed three major clusters: (1) clinical practice guidelines and therapeutic recommendations, (2) pharmacokinetic and pharmacodynamic studies, and (3) emerging research on antifungal resistance mechanisms. This structure indicates that the field has evolved from foundational pharmacological and clinical guidance toward addressing resistance challenges, mirroring the keyword evolution trends.

### Additional bibliometric indicators

The overall dataset n = 47 had a total of 645 citations, with an average of 13.7 citations per publication and an H-index of 16. The citation density (average citations per year) was 32.3 (Geerlings, 2016). The most influential journals (**Table 4**) were Clinical Infectious Diseases (impact factor 11.8, 2 publications), Antimicrobial Agents and Chemotherapy (IF 5.2, 3 publications), and World Journal of Urology (IF 3.8, 1 publication), reflecting the multidisciplinary nature of this research area.

**Table 4:** Top 10 most influential journals publishing echinocandin-UTI research.

Rank	Journal	Publications	Total citations	Average citations per publication	Impact factor
1	Clinical Infectious Diseases	2	201	100.5	11.8
2	Antimicrobial Agents and Chemotherapy	3	62	20.7	5.2
3	World Journal of Urology	1	50	50.0	3.8
4	Journal of Clinical Microbiology	1	48	48.0	6.1
5	Expert Review of Anti-infective Therapy	1	31	31.0	4.7
6	Antibiotics	1	31	31.0	4.7
7	International Journal of Antimicrobial Agents	1	25	25.0	4.9
8	Canadian Journal of Infectious Diseases and Medical Microbiology	1	25	25.0	2.2
9	Medical Mycology	1	24	24.0	3.6
10	Acta Clinica Belgica	1	17	17.0	2.1

## Discussion

### Clinical effectiveness of echinocandins in UTIs

Urinary tract infections (UTIs) are common among hospitalized patients, with fungal UTIs predominantly caused by Candida species (Lodise et al., 2021; Chaari et al., 2023; Konje et al., 2023; Castellano-Sánchez et al., 2025). Current guideline-recommended antifungal therapies include fluconazole, amphotericin B deoxycholate, and flucytosine (Alothman et al., 2014; Pappas et al., 2016). However, accumulating evidence suggests that echinocandins may represent a safe and effective alternative for selected cases of urinary Candida infections.

The therapeutic efficacy of echinocandins in UTIs appears to depend primarily on renal tissue penetration rather than urinary drug excretion. Although urinary excretion rates are extremely low (<2%), pharmacokinetic studies and animal models have demonstrated that caspofungin and micafungin rapidly penetrate renal parenchymal tissue and achieve concentrations exceeding the minimum inhibitory concentrations (MICs) for most clinically relevant Candida species (Eschenauer et al., 2007; Howard et al., 2011; Bellmann and Smuszkiewicz, 2017). These findings support the hypothesis that sufficient tissue exposure may enable effective antifungal activity in upper urinary tract infections, including pyelonephritis and deep bladder wall infections (Rezai et al., 2017).

Importantly, this pharmacokinetic profile has differential implications for simple cystitis versus complicated upper UTIs. In uncomplicated lower UTIs, the extremely low urinary excretion renders echinocandins suboptimal, as achieving adequate concentrations in the bladder lumen is the primary determinant of efficacy. In contrast, for pyelonephritis and complicated UTIs with renal parenchymal involvement, tissue penetration becomes the critical factor, and echinocandins may offer a therapeutic advantage despite low urinary concentrations.

Further pharmacodynamic evidence supports their potential clinical utility. Grau et al. reported that a urinary peak concentration-to-MIC ratio greater than 4 for micafungin was associated with treatment success, providing a mechanistic rationale for its application in clinical practice (Grau et al., 2016). The validation and refinement of such PK/PD targets represent a critical frontier for future research, particularly as MICs continue to rise in multidrug-resistant isolates such as C. auris and C. glabrata.

### Safety profile of echinocandins

Echinocandins have consistently demonstrated favorable safety profiles in the treatment of invasive candidiasis, and similar findings have been reported in urinary tract infection (UTI)-related applications (Thompson et al., 2023). Overall, serious treatment-related adverse events appear to be uncommon, supporting their potential use as a well-tolerated antifungal option in selected patients.

Evidence from clinical studies further supports this safety profile. The MSG-01 study reported only one serious drug-related adverse event among 219 high-risk intensive care unit patients receiving caspofungin prophylaxis (Ostrosky-Zeichner et al., 2014). Similarly, case series evaluating echinocandin therapy for candiduria have demonstrated excellent tolerability, with no severe hepatotoxicity or treatment discontinuation attributable to adverse effects (Rkieh et al., 2022).

The most commonly reported adverse events include transient elevations in liver enzyme levels, infusion-related reactions, mild gastrointestinal symptoms, headache, and phlebitis. These events are generally mild, reversible, and manageable with appropriate clinical monitoring.

### Comparative analysis of echinocandins and fluconazole

Fluconazole remains the preferred first-line therapy for candiduria because of its excellent urinary penetration, high oral bioavailability, and low cost (Rabeneck and Laine, 1994; Wildfeuer et al., 1996; Pappas et al., 2016). However, the increasing prevalence of azole resistance among non-albicans Candida species has renewed clinical interest in alternative antifungal strategies, particularly echinocandins.

Current comparative evidence suggests that echinocandins may provide comparable therapeutic efficacy in selected patient populations, particularly those with fluconazole-resistant infections, severe systemic illness, renal parenchymal involvement, or Candida glabrata infection. These clinical scenarios highlight the potential advantages of echinocandins when conventional azole therapy is limited by resistance or inadequate tissue penetration.

Supporting this perspective, the multicenter propensity score-matched study conducted by Cuervo et al. demonstrated no significant differences between echinocandins and fluconazole with respect to 30-day mortality or treatment success rates (Cuervo et al., 2017). These findings suggest that echinocandins may represent a viable alternative to fluconazole in appropriately selected patients.

### Rezafungin: a next-generation echinocandin with distinctive pharmacokinetics

Rezafungin is a novel semisynthetic echinocandin with a distinctive pharmacokinetic profile that distinguishes it from other agents in its class. Unlike caspofungin, micafungin, and anidulafungin, rezafungin exhibits long-acting pharmacokinetics with a half-life of approximately 130–133 hours, enabling once-weekly intravenous dosing (Sandison et al., 2017). This prolonged half-life is attributable to its high stability and slow elimination, with approximately 38% of the dose recovered in feces and 14% in urine (Ong et al., 2022).

The urinary excretion of rezafungin (approximately 14%) is substantially higher than that of other echinocandins (<2%), suggesting potentially greater utility in UTIs. Furthermore, rezafungin has demonstrated potent *in vitro* activity against a broad range of Candida species, including fluconazole-resistant and echinocandin-resistant isolates (Locke et al., 2025).

Accumulating case reports have demonstrated successful outcomes with echinocandin therapy in patients with candiduria, particularly those who had failed prior fluconazole or amphotericin B therapy due to azole resistance or underlying hepatic and renal impairment. In these challenging scenarios, the achievement of a high Cmax/MIC ratio appears to be a critical determinant of therapeutic success, as echinocandins can eliminate over 99% of the renal fungal burden despite low urinary concentrations (Lepak et al., 2018). This pharmacodynamic principle may also extend to rezafungin, given its potent activity and favorable tissue penetration profile.

The once-weekly dosing schedule of rezafungin offers several potential advantages for UTI management: (1) reduced intravenous access requirements, (2) potential for outpatient parenteral antimicrobial therapy (OPAT), (3) improved patient convenience and adherence, and (4) reduced healthcare costs associated with daily infusions (Forrister et al., 2025). However, robust clinical data specifically evaluating rezafungin for UTIs remain limited, and its role in this setting requires further investigation.

### Antimicrobial stewardship implications

The emergence of C. auris and C. glabrata as research hotspots in our bibliometric analysis reflects a broader global phenomenon: the escalating prevalence of azole resistance among Candida species. This resistance has direct implications for antifungal stewardship, as the increasing reliance on echinocandins for UTIs is not merely a clinical curiosity but a direct consequence of diminishing therapeutic options.

The IDSA guidelines currently discourage routine echinocandin use for candiduria, yet emerging evidence—including the cases identified in our bibliometric analysis—suggests that echinocandins may be necessary in specific scenarios: (1) fluconazole-resistant C. glabrata infections, (2) C. auris infections with intrinsic azole resistance, (3) severe systemic illness with renal parenchymal involvement, and (4) failure of conventional therapy.

From a stewardship perspective, this creates a tension between guideline recommendations and clinical necessity. We advocate for updated, evidence-based stewardship protocols that: (1) clearly define indications for echinocandin use in UTIs, (2)Establish criteria for therapeutic drug monitoring of echinocandins to guide the treatment of urinary tract infections, (3) promote judicious use to preserve echinocandin efficacy, and (4) monitor for emergence of echinocandin resistance.

### Comparison with previous bibliometric studies

Our findings align with previous bibliometric analyses in related fields. For instance, bibliometric studies on invasive candidiasis have similarly identified antifungal resistance and C. auris as emerging hotspots, reflecting global epidemiological trends. However, the total number of publications in our study *n* = 47 is substantially smaller than that in broader Candida or antifungal resistance bibliometric analyses, which typically include hundreds or thousands of publications. This discrepancy underscores the niche and emerging nature of echinocandin-UTI research specifically. Compared with bibliometric analyses on bacterial UTIs, our study shows that fungal UTIs, and particularly echinocandin therapy, remain under-represented, highlighting a knowledge gap that merits greater research investment.

### Study limitations and future directions

This study has several limitations. First, the literature search was limited to PubMed and Web of Science; other databases such as Scopus and Embase were not included. This decision was primarily based on the fact that Web of Science and PubMed represent the gold-standard data sources for bibliometric analyses, offering the most comprehensive citation data and optimal compatibility with CiteSpace and VOSviewer. Given that the total number of publications in this niche field was already limited (47 articles), these two core databases were sufficient to delineate the global collaboration networks and hotspot evolution. Nevertheless, we acknowledge that the exclusion of Scopus/Embase may have resulted in the omission of a small number of non-indexed case reports or regional studies, and we have addressed this as an objective limitation. Second, only English-language publications were included, potentially introducing language bias. Third, bibliometric analyses are inherently dependent on the accuracy and completeness of database metadata, potentially affecting the reliability of network construction. Fourth, the relatively limited number of included studies may have influenced the stability of the bibliometric networks. Fifth, citation-based indicators may be affected by citation lag, particularly for recently published articles (2025–2026), and by self-citation, which we did not separately analyze. Sixth, the heterogeneity of publication types (reviews, case reports, original research) may introduce citation bias, as review articles generally receive higher citation counts than primary studies. These limitations should be considered when interpreting our findings.

Future research should focus on: (1) conducting large-scale randomized controlled trials to establish definitive efficacy and safety; (2) validating PK/PD parameters, particularly urinary Cmax/MIC ratios, to guide dosing; (3) evaluating the clinical potential of rezafungin for UTIs, including outpatient and step-down therapy; (4) strengthening antifungal resistance surveillance, especially for C. auris and C. glabrata; and (5) performing pharmacoeconomic assessments to inform stewardship protocols and guideline updates.

## Conclusion

This bibliometric analysis systematically characterized the research landscape and developmental trajectory of echinocandin therapy for urinary tract infections (UTIs). Although annual publication output remains relatively limited, sustained scholarly attention highlights the growing clinical relevance of this field. Research hotspots have progressively shifted from conventional candiduria management toward multidrug-resistant fungal pathogens, particularly Candida auris and Candida glabrata, reflecting an important evolution in clinical focus from assessing the feasibility of echinocandin use in UTIs to optimizing their integration into the management of resistant fungal urinary infections.

The discrepancy between current IDSA guidelines, which discourage routine echinocandin use, and accumulating clinical evidence suggesting efficacy in selected scenarios highlights the urgent need for updated, evidence-based stewardship protocols. Specifically, we advocate for the development of clinical algorithms that clearly define indications for echinocandin use in fluconazole-refractory UTIs, establish criteria for antifungal susceptibility testing to guide therapeutic decisions, and promote judicious use to preserve echinocandin efficacy while minimizing the emergence of resistance. Robust prospective clinical studies, including randomized controlled trials and pharmacokinetic/pharmacodynamic evaluations, remain essential to establish the definitive therapeutic role of echinocandins in this setting and to inform evidence-based guideline updates.

## Data Availability

The original contributions presented in the study are included in the article/**Supplementary Material**. Further inquiries can be directed to the corresponding author.
