# Supplementary material for: A bibliometric and visual analysis of research on echinocandin treatment for urinary tract infections based on VOSviewer and CiteSpace
Source: Front Cell Infect Microbiol. 2026 Sep 22;16:1891369. doi: 10.3389/fcimb.2026.1891369 (PMC13638403; doi:10.3389/fcimb.2026.1891369)
Supplement: Supplementary file 1 [file SupplementaryFile1.docx]

**Table 1.** Search Strategy for Identifying English Literature on Echinocandins for Urinary Tract Infections

| **Database** | **Search Strategy** |
| --- | --- |
| **PubMed** | ("Echinocandins"[MeSH Terms] OR "Echinocandins/therapeutic use"[MeSH Terms] OR "Caspofungin"[MeSH Terms] OR "Caspofungin/therapeutic use"[MeSH Terms] OR "Micafungin"[MeSH Terms] OR "Micafungin/therapeutic use"[MeSH Terms] OR "Anidulafungin"[MeSH Terms] OR "Anidulafungin/therapeutic use"[MeSH Terms] OR "Rezafungin"[Supplementary Concept] OR echinocandin*[Title/Abstract] OR caspofungin*[Title/Abstract] OR micafungin*[Title/Abstract] OR anidulafungin*[Title/Abstract] OR rezafungin[Title/Abstract] OR ibrexafungerp[Title/Abstract]) AND ( "Urinary Tract Infections"[MeSH Terms] OR "Urinary Tract Infections/drug therapy"[MeSH Terms] OR "Pyelonephritis"[MeSH Terms] OR "Pyelonephritis/drug therapy"[MeSH Terms] OR "Cystitis"[MeSH Terms] OR "Cystitis/drug therapy"[MeSH Terms] OR "Bacteriuria"[MeSH Terms] OR "Pyuria"[MeSH Terms] OR "urinary tract infection*"[Title/Abstract] OR "UTI"[Title/Abstract] OR "urinary infection*"[Title/Abstract] OR "bladder infection*"[Title/Abstract] OR "cystitis"[Title/Abstract] OR "pyelonephritis"[Title/Abstract] OR "bacteriuria"[Title/Abstract] OR "pyuria"[Title/Abstract] OR "catheter-associated urinary tract infection*"[Title/Abstract] OR "CAUTI"[Title/Abstract]) |
| **Web of Science** | TI=(echinocandin* OR "caspofungin" OR "micafungin" OR "anidulafungin" OR "rezafungin") AND TI=("urinary tract infection*" OR "UTI" OR "urinary infection*" OR "bladder infection*" OR "cystitis") OR AB=((echinocandin* OR "caspofungin" OR "micafungin" OR "anidulafungin" OR "rezafungin") AND ("urinary tract infection*" OR "UTI" OR "urinary infection*" OR "bladder infection*" OR "cystitis")) |
