# Supplementary material for: A bibliometric and visual analysis of research on echinocandin treatment for urinary tract infections based on VOSviewer and CiteSpace
Source: Front Cell Infect Microbiol. 2026 Sep 22;16:1891369. doi: 10.3389/fcimb.2026.1891369 (PMC13638403; doi:10.3389/fcimb.2026.1891369)
Supplement: Supplementary file 2 [file SupplementaryFile2.docx]

**Table 2.** Search Parameters Used to Generate VOSviewer Visualization Maps

| **Figure** | **Search Parameters** |
| --- | --- |
| Figure 3 | Data sources: PubMed and Web of Science. Access date: March 20, 2026. The analysis included 47 eligible articles published from database inception to March 20, 2026. VOSviewer was used to visualize collaboration networks among institutions. The threshold was set to 2. |
| Figure 4 | Data sources: PubMed and Web of Science. Access date: March 20, 2026. The analysis included 47 eligible articles published from database inception to March 20, 2026. VOSviewer was used to visualize collaboration networks among authors. The threshold was set to 17. |
| Figure 5 | Data sources: PubMed and Web of Science. Access date: March 20, 2026. The analysis included 47 eligible articles published from database inception to March 20, 2026. VOSviewer was used to visualize co-occurrence networks of keywords. The threshold was set to 2. |
